# Enhancing future acceptance of rural placement in Tanzania through peripheral hospital rotations for medical students

**DOI:** 10.1186/s12909-016-0582-8

**Published:** 2016-02-09

**Authors:** Gibson Erick Kapanda, Charles Muiruri, Ahaz T. Kulanga, Chrispina N. Tarimo, Esther Lisasi, Lucy Mimano, Kien Mteta, John A. Bartlett

**Affiliations:** Kilimanjaro Christian Medical University College, Moshi, Tanzania; Duke Global Health Institute, Durham, NC USA; Duke University School of Medicine, Durham, NC USA

**Keywords:** Peripheral hospital clerkship, Rural practice, Third year medical students

## Abstract

**Background:**

Mal-distribution of health care workers is a global health challenge that exacerbates health disparities, especially in resource-limited settings. Interventions to mitigate the problem have targeted qualified personnel with little focus on medical students. However, studies have demonstrated that rural rotations positively influence students to practice in rural settings upon graduation. To evaluate the influence of peripheral rotations in a resource-limited setting, the Kilimanjaro Christian Medical University College introduced a 12-week clerkship rotation in peripheral hospitals for third-year medical (MD3) students in 2012. We administered an end-of-rotation survey to assess student perceptions, and attitudes toward rural practice after graduation.

**Methods:**

Questionnaires were voluntarily and anonymously administered to MD3 students in April 2014. The questions assessed perceptions of the experience, and attitudes towards rural practice upon graduation. The perceptions were assessed using strength of consensus measures (sCns). The effect of the experience on likelihood for rural practice was assessed using Crude Odds Ratio (COR), and predictors using Adjusted Odds Ratio (AOR) with 95 % Confidence Intervals (CI) tested at a 5 % level of significance. Variation was assessed using Hosmer and Lemeshow test Chi-square.

**Results:**

111 out of 148 MD3 students participated; 62 % were male; 62 % <25 years old; and 72 % matriculated directly from secondary school. Overall, 81 % of MD3 students were satisfied with rural rotations (sCns = 83 %). The likelihood of accepting rural practice deployment after graduation was predicted by satisfaction with the peripheral hospital rotation program (AOR, 4.32; 95 % CI, 1.44–12.96; *p*, 0.009) and being male (AOR, 2.73; 95 % CI, 1.09–6.84; *p*, 0.032). Students admitted in medical school after health-related practice trended toward a higher likelihood of accepting rural practice after graduation compared to those enrolled directly from secondary school, although the difference was not significant (AOR, 4.99; 95 % CI, 0.88–28.41; *p*, 0.070). The Hosmer and Lemeshow test *p*-value was 0.686, indicating a good fit of the model. No significant differences in satisfaction between these two groups were observed, and also no significant differences between students born in rural areas compared to those born in urban areas existed.

**Conclusion:**

Results indicate that satisfaction with rural rotations is associated with increased likelihood of rural practice after graduation. We conclude that opportunities may exist to reduce mal-distribution of healthcare workers through interventions that target medical students.

## Background

Mal-distribution of health care workers is a global health challenge that exacerbates health disparities [1–3]. This mal-distribution is especially pronounced in resource-limited settings where inequalities in infrastructure between urban and rural areas exist [1, 3]. For example, a country like Tanzania faces high levels of imbalance in physician distribution between rural and urban areas. The numbers of physicians and specialists (consultants) per 10,000 population ratio are estimated at 0.260 and 0.079 respectively [4], which is far below the recommended ratio of 1:5,000 [5]. In 2013, of all registered physicians in Tanzania, 69 % were serving in the urban areas and only 31 % were serving rural populations, where 75 % of Tanzanians resided [6]. These healthcare inequities have significant impact on access and quality of healthcare in areas of great need [7].

Complex and overlapping factors have been related to these disparities in human resources for health. These factors are largely associated with health professional’s personal characteristics and preferences, health systems, and the social, political and economic environments [8]. Poor working conditions (including poor facilities such as equipment and infrastructure, and lack of drugs and supplies), limited future career prospects, and inadequate social amenities have been postulated as factors deterring health workers from choosing and remaining in rural practice [9].

Several interventions have attempted to reduce the urban-rural health workforce imbalances through recruitment and retention of health workers in rural and underserved areas. According to the World Health Organization, such interventions have included increasing the number of students enrolled in medical and allied sciences schools, regulatory policies such as compulsory rural service, monetary incentives or bonuses, access to continuing medical education opportunities, and provision of non-monetary incentives such as preferential selection of students for under- and post-graduate studies from rural areas [8]. However, the results of these interventions and their impact have been modest, including the challenges of sustainability [8]. For example, despite the implementation of most of the above-mentioned interventions, the proportion of practicing physicians in rural Tanzania has remained the same [6, 10–12]. These disparities also still exist despite an almost five-fold increase in the annual medical school enrolment in both public and private universities in Tanzania since 1997 [13]. Despite the increase in enrolment, there has been a dearth of interventions that target medical students to increase the recruitment and retention of these future health care workers into rural areas. Current resources for interventions have largely targeted qualified personnel [14]. Interventions that target medical students may have an effect on future choice of practice location and eventual retention in these areas. Wilson and colleagues found that interventions targeting medical schools could mitigate the rural health workforce shortages [15]. For example, there is evidence that training medical students with rural backgrounds (born and/or brought up in rural areas) and provision of medical training in schools located in rural areas could result in choice of rural medical practice after graduation. [16–21]. Also, another recent study in Australia found that rural background and/or training in a rural medical school predicted uptake of rural medical practice. As such, they found strong interaction between rural background and rural exposure during undergraduate training in predicting future rural medical practice [22]. Some studies have found that personal characteristics of medical students may influence acceptance of positions in rural hospitals. Age of a health professional may affect placement decisions [23]; for example, Leon and Kolstad found that age above 26 years increased the likelihood of accepting job offers in a rural hospital [24]. However, inconclusive findings have been reported on the influence of gender on acceptance of deployment to rural hospitals. For example, Wilkinson et al. [20] found a predominance of male general practitioner doctors in rural areas of Australia, implying increased likelihood of male acceptance of rural practice. Similarly, Kotha reported increased preference of male medical students for rural practice in Ghana [25]. Mehboob et al. did not find significant gender differences on preference for rural medical career in Saudi Arabia [26]. The argument for this difference could partly be explained by differences in family responsibilities between male and female, which differ among countries and among communities in the same country [7].

Most of the medical schools in sub-Saharan Africa, including Tanzania, are located in urban centers and have concentrated their clinical clerkships in tertiary hospitals, where curative and highly specialized medical education is emphasized [27, 28]. Little emphasis has been put on community-based medical education, which has been shown to predict future intention for rural medical practice after graduation [29, 30]. Admission of medical students from rural areas has proved futile due to the fact that most secondary schools in rural areas are ill-equipped with laboratory spaces and lack science teachers to prepare them for admission in medical schools [31]. Furthermore, poor infrastructure in rural areas (lack of electricity, poor roads, poor communication services, and general poverty of rural populations) hampers the establishment of medical schools in rural settings, and as a consequence most medical schools are located in urban centers [32].

Rural clinical placements have been shown to have positive influence on medical student choices for rural practice after graduation. For example, Schoo et al. found that rural placement of Australian medical students during training was associated with starting practice in rural areas after graduation [33]. Also, a case–control study in South Africa demonstrated that rural exposure influenced the choice of practice. However, authors were skeptical about the influence of historical developments specific to South Africa such as apartheid which were not studied [29]. A qualitative study in South Africa [34] demonstrated that the decision of health care professionals for rural practice is enhanced by rural exposure during training, awareness of rural needs and influence of rural role models.

Studies have further reported that rural placements during training must provide a positive experience, which include good supervision and support in order to increase the likelihood of returning to rural areas [35]. Also, Rourke argued that the provision of positive rural learning experiences in medical schools could increase the number of graduating physicians with an interest in rural practice [17]. In a systematic literature review on the effectiveness of preceptors for medical students, the authors found that durations as short as 3 weeks influenced student career choices when preceptors were rated as quality teachers [21, 36]. Specifically, the authors emphasized the role of positive undergraduate experiences with preceptors during rural placements on the uptake of rural practice after medical school [21, 36]. Many of these studies addressing the influence of rural rotations on preferences for rural practice after graduation have been conducted in developed countries (Australia, Canada, and USA) and to some extent in South Africa (a medium-income country), but none have been conducted in a low-income sub-Saharan Africa country.

With the aim of exposing medical students to rural working environments, in 2012 the Kilimanjaro Christian Medical University College (KCMUCo) introduced 12-week peripheral hospital rotations into the junior clerkship curriculum to third-year medical (MD3) students. This was undertaken in part to address congestion during clinical training at the Kilimanjaro Christian Medical Centre (KCMC), and also to expose the students to the rural working environments. Eight hospitals had been carefully selected for this purpose (Table 1) after initial assessment of their appropriateness for training MD3 students during their junior clerkship. The selected hospitals had to demonstrate ability to accommodate students and adequate supervisors for students. Preceptors from these hospitals received adjunct appointments at KCMUCo and specialists from KCMC provided weekly clinics at the hospitals during the rotation period. This intervention was part of a larger project entitled the Medical Education Partnership Initiative (MEPI), which was conducted in collaboration with Duke University School of Medicine (DUSOM) and funded by the United States Government (USG). One of the main goals of the MEPI program was to develop interventions that would enhance retention of health care workers in areas of great need such rural locations. The survey, therefore, aimed to assess the likelihood and the factors that would influence acceptance of rural practice upon graduation among students who underwent short-term peripheral hospital clinical clerkship exposure during medical training.

**Table 1:** Peripheral hospital characteristics

S/N	Health facility name	Number of students	Doctors available					Total	Official bed capacity	Average daily bed occupancy	Average daily OPD attendance	Distance (km)
			Specialists									
			Obs tetrics & gynaecology	Paediatrics	Surgery	Internal medicine	General practitioners					
1	Mawenzi regional referral hospital	15	0	0	0	0	0	0	300	164	97	5
2	St. Joseph designated district hospital	7	0	0	1	0	6	7	150	140	200	8
3	Kibosho designated district hospital	17	0	0	1	0	4	5	180	60	200	10
4	St. Elizabeth hospital	12	0	0	0	0	4	4	100	87	90	80
5	Mt. Meru regional referral hospital	27	3	3	3	0	12	18	500	200	180	80
6	Tanganyika Planting Company (TPC) hospital	15	0	0	0	0	1	1	90	65	200	18
7	Same district hospital	10	0	0	0	0	4	4	136	70	80	105
8	Gonja hospital	8	0	0	0	0	3	3	67	50	50	165

## Methods

### Study design

Non-experimental.

A cross-sectional survey was administered to students who participated in the peripheral hospital rotation.

### Study population and sampling

Study population included all third year medical students at KCMUCo. Since the peripheral hospital junior clerkship rotation is a compulsory curriculum activity, all third year students were eligible to participate in the study. A census was thus employed for students who were willing and available to participate in the survey.

### Data collection methods

A self-administered questionnaire was used to collect data from students soon after their return from the peripheral hospital rotation in April 2014. The questions assessed students’ perceptions of the experience, attitudes towards the peripheral hospital rotation and willingness to accept rural practice upon graduation. The perceptions and attitude were assessed using 5-point Likert scale (strongly agree”5” to strongly disagree “1”).

### Reliability

Since no standardized and validated questionnaires were available, the team developed a questionnaire that was piloted to 10 fourth year medical students (MD4) who had done the peripheral hospital junior clinical clerkship rotation the previous year and showed high reliability with Cronbach’s Alpha of 0.949.

### Ethical issues

Since this survey was part of the monitoring and evaluation activities of the MEPI project, and hence educational, ethical approval was exempted by the Kilimanjaro Christian Medical University College Research Ethics Committee and consent was sought from students before completing the questionnaires after explanation of the purpose and benefits of the survey. Permission for withdrawal from participation in the survey without any penalties was explained to the students. No names appeared on the questionnaires; instead code numbers were used. Thus, the survey was voluntary and anonymous.

### Data processing and analysis

Data entry was completed using IBM Statistical Package for Social Sciences (SPSS) version 20.0 computer program. Strength of consensus measure (sCns) was used to measure the strength of agreement/disagreement to statements since it objectively assesses the level of group/team agreement to a construct. A strength of consensus ≥80 % was considered significantly strong. The effect of the peripheral hospital clerkship experience on likelihood for acceptance of rural practice after graduation was assessed using Crude Odds Ratio (COR) with 95 % Confidence Intervals (CI), and tested with a 5 % level of significance. Binary logistic regression analysis was used to determine predictors of accepting rural practice after graduation with Adjusted Odds Ratio (AOR) with 95 % CI, and the Hosmer and Lemeshow Test was used to test goodness-of-fit of the model.

## Results

**Fig. 1:**
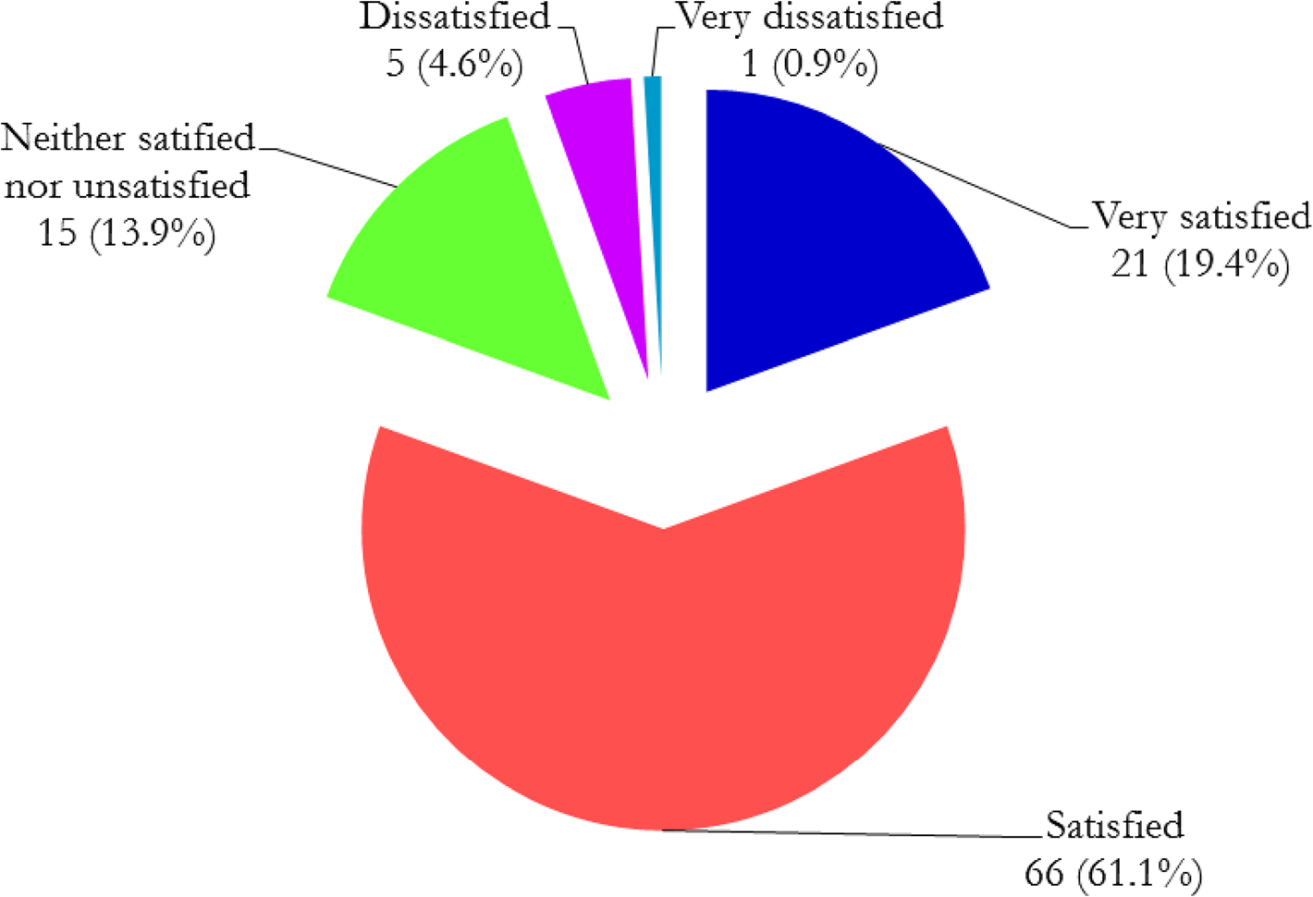
Overall satisfaction with peripheral hospital placement program

**Fig. 2:**
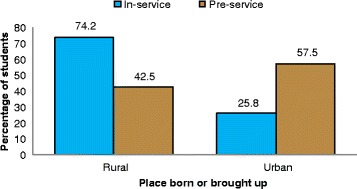
Students’ status at enrolment according to place born or brought up

Of 148 MD3 students, 111 (75 %) responded to the survey; 62 % were male, 62 % younger than 25 years and 28 % enrolled after health-related practice in a junior cadre. Figure 1 shows the overall satisfaction of MD3 students with the peripheral hospital rotations. More than 80% of MD3 were either satisfied (61.1 %) or very satisfied (19.4 %) with the peripheral hospital placement. Only 5.5 % of the students were either dissatisfied (4.6 %) or very dissatisfied (0.9 %) with their peripheral hospital placements. The majority of the dissatisfied students had been born or brought up in urban areas, were male, and joined medical school directly from secondary school. All of them were government-sponsored students. About three quarters (74 %) of students enrolled after health-related practice (in-service) had been born or brought up in rural areas as compared to 43 % students enrolled direct after completing secondary education (pre-service) (Fig. 2). In-service students were about 4 times significantly more likely to have been born or brought up in rural areas than pre-service (OR, 3.9; 95 % CI, 1.6–9.7; *p*, 0.003).

Further analysis was done using the Strength of Consensus (sCns) measure to assess the aspects for which students were satisfied (Table 2). Overall, 81 % of MD3 students were satisfied with rural rotations (mean sCns = 83 %). Most students had favorable perceptions with significant sCns measures ranging from 83.5 to 86.5 %. A weak strength of consensus measure (74.4 %) was noted only for the statement ‘Easy to practice clinical skills in the peripheral hospital’.

**Table 2:** Satisfaction with aspects of peripheral hospital placement

Satisfaction with clinical practice in a peripheral hospital	*n*	SA	A	*N*	Dis	SD	Mean (SDev)	sCns (%)
		*n* (%)	*n* (%)	*n* (%)	*n* (%)	*n* (%)		
Easy to practice clinical skills in the peripheral hospital	110	19 (17.3)	58 (52.7)	24 (21.8)	6 (5.5)	3 (2.7)	3.8 (0.9)	74.4
Learnt a lot on clinical clerkship skills in a peripheral hospital	110	40 (36.4)	57 (51.8)	9 (8.2)	2 (1.8)	2 (1.8)	4.2 (0.8)	83.6
Would want to return to practice clinical skills at a peripheral hospital	110	46 (41.8)	46 (41.8)	13 (11.8)	3 (2.7)	2 (1.8)	4.2 (0.9)	83.4
Glad that I had the opportunity to utilize my clinical skills at a peripheral hospital	110	47 (42.7)	54 (49.1)	6 (5.5)	1 (0.9)	2 (1.8)	4.3 (0.8)	85.8
Enjoyed practicing at a peripheral hospital	110	40 (36.4)	54 (49.1)	13 (11.8)	2 (1.8)	1 (0.9)	4.2 (0.8)	83.5
I can now see the importance of basic sciences in my clinical practice	110	50 (45.5)	50 (45.5)	7 (6.4)	2 (1.8)	1 (0.9)	4.3 (0.8)	86.5
Worthwhile teaching clinical practice in a peripheral hospital	110	41 (37.3)	55 (50.0)	9 (8.2)	4 (3.6)	1 (0.9)	4.2 (0.8)	83.6
Mean							4.2	83.0

*n* number of students, *SA* Strongly Agree, *A* Agree, *N* Neutral/Undecided, *Dis* Disagree, *SD* Strongly Disagree, *SDev* Standard Deviation, *sCns* Strength of consensus measure

Furthermore, students were asked to indicate their attitudes towards future peripheral hospital placements. Overall, 84 % of students exhibited positive attitudes toward peripheral hospital placements (mean sCns = 80.4 %) (Table 3). Specific attitudes on peripheral hospital placements had sCns ranging from 81.8 to 83 %, with the exception of the statement ‘I am very likely to accept deployment to the rural hospitals after completion of my studies’, which exhibited a weak strength of consensus measure (sCns = 73 %).

**Table 3:** Attitudes toward peripheral hospital placement

Attitude towards peripheral hospital placement	*n*	SA	A	*N*	Dis	SD	Mean (SDev)	sCns (%)
		*n* (%)	*n* (%)	*n* (%)	*n* (%)	*n* (%)		
I like the idea of peripheral hospital placement	108	29 (26.9)	67 (62.0)	8 (7.4)	2 (1.9)	2 (1.9)	4.1 (0.8)	81.9
I have general favourable attitude towards peripheral hospital placement	107	30 (28.0)	62 (57.9)	13 (12.1)	0 (0.0)	2 (1.9)	4.1 (0.8)	81.9
I believe that it is a good idea to place students in the peripheral hospital in clinical care	108	35 (32.4)	61 (56.5)	7 (6.5)	2 (1.9)	3 (2.8)	4.1 (0.8)	82.4
Peripheral hospital placement is a great idea	108	35 (32.4)	60 (55.6)	10 (9.3)	1 (0.9)	2 (1.9)	4.2 (0.8)	83.0
I am very likely to accept deployment to the rural hospitals after completion of my studies	108	19 (17.6)	53 (49.1)	27 (25.0)	4 (3.7)	5 (4.6)	3.7 (1.0)	73.0
Mean							4.0	80.4

*n* number of students, *SA* Strongly Agree, *A* Agree, *N* Neutral/Undecided, *Dis* Disagree, *SD* Strongly Disagree, *SDev* Standard Deviation, *sCns* Strength of consensus measure

Bivariate and multivariate logistic regression analyses of the determinants of acceptance of rural hospital placements after graduation are shown in Table 4. Bivariate analysis of student characteristics and acceptance of deployment to a rural hospital after graduation demonstrated a significantly higher likelihood if the respondent’s gender was male (COR, 3; 95 % CI, 1.3–7.0; *p* = 0.008), age 25 years or above (COR, 2.8; 95 % CI,1.1–7.0; *p*,0.024), enrolment after health-related practice (in-service) (COR, 6.2, 95 % CI,1.7–22.3; *p*, 0.002), born in a rural area (COR, 2.5, 95 % CI,1.1–5.7; *p*,0.029), and being satisfied with peripheral hospital placement (OR,4.5; 95 % CI,1.7–12.3; *p*, 0.002).

**Table 4:** Students’ characteristics and willingness to accept a job in a rural hospital

Variable	Total	Acceptance of deployment to a rural hospital		COR (95 % CI)	AOR (95 % CI)	*p*-value
		Will accept	Will not accept			
		No. (%)	No. (%)			
*Sex:*						
Male	67	51 (76.1)	16 (23.9)	3.0 (1.3–7.0)	2.7 (1.1–6.8)	0.032
Female	41	21 (51.2)	20 (48.8)	1.0	1.0	
*Age (years):*						
25 or older	40	32 (80.0)	8 (20.0)	2.8 (1.1–7.0)	0.9 (0.2–3.5)	0.898
Younger than 25	68	40 (58.8)	28 (41.2)	1.0	1.0	
*Enrolment status:*						
In-service	29	26 (89.7)	3 (10.3)	6.2 (1.7–22.3)	5.0 (0.9–28.4)	0.070
Pre-service	79	46 (58.2)	33 (41.8)	1.0	1.0	
Place of birth and/or upbringing:						
Rural	55	42 (76.4)	13 (23.6)	2.5 (1.1–5.7)	1.8 (0.7–4.7)	0.214
Urban	53	30 (56.6)	23 (43.4)	1.0	1.0	
Satisfaction with rural placement:						
Satisfied	87	64 (73.6)	23 (26.4)	4.5 (1.7–12.3)	4.3 (1.4–13.0)	0.009
Not satisfied	21	8 (38.1)	13 (61.9)	1.0	1.0	

Multivariate logistic regression revealed that the likelihood of accepting deployment in rural practice after graduation was predicted by being satisfied with the peripheral hospital rotation program (AOR, 4.32; 95 % CI, 1.44–12.96; *p*, 0.009) and being male (AOR, 2.73; 95 % CI, 1.09–6.84; *p*, 0.032). Also, enrolment after health-related practice increased the likelihood of accepting rural practice after graduation by 300 % compared to enrolment direct from secondary school, although the difference was not significant (AOR, 4.99; 95 % CI, 0.88–28.41; *p*, 0.070). The Hosmer and Lemeshow test Chi-square was 3.9 with p-value 0.686, indicating a good fit of the model, that is, our model predicts values not significantly different from what we observed.

## Discussion

Interventions aimed at retaining healthcare providers in settings of high need have been focused on qualified individuals, and despite these efforts, there has been minimal effect. There is a dearth of interventions that focus on medical students or those in in-service training. Studies looking at factors that influence acceptance of rural practice post training of medical students have illuminated the need to conduct interventions that will expose students to rural placements. This survey found that, for the MD3 students who responded to our survey, the majority were satisfied with the peripheral hospital rotation with a strong strength of consensus measure. Also, majority of students had positive attitudes toward peripheral hospital placements as indicated by a significant strength of consensus measure. However, weak strength of consensus measures were noted for ease to practice clinical skills at the peripheral hospital, and attitudes towards undertaking rural practice after graduation.

Our study found strong consensus (sCns > 83 %) in gaining clinical clerkship skills after getting an opportunity to utilize these skills at the peripheral hospital. This finding corroborates the observations of Kibore et al. in Kenyan students who reported satisfaction after a 7-week peripheral hospital rotation. Specifically, they felt they had taken an active role in patient care, had improved their clinical skills, and had learned to tackle socio‑cultural challenges in patient care [37]. Other authors elsewhere in the developed world also observe high satisfaction after a rural rotation, due to factors such as seeing a wide variety of patients [38, 39], and thus perceiving their rural experiences as an important component of medical education [40].

A majority of students indicated their willingness to accept deployment in a peripheral (rural) hospital after graduation. Other studies have also shown that peripheral rotations, when well organized and with good clinical and personal support to students, can positively influence students’ attitudes toward rural practice [8, 24, 37]. Though, Couper [41] acknowledges that the influence of stand-alone short-term rural placements on practice choices can be strengthened if integrated by other interventions, evidence suggests that placements as short as 4–6 weeks during training can positively influence rural medical practice choice [42].

Bivariate analysis of the factors influencing student willingness to accept deployment to a rural hospital after graduation demonstrated that age above 25 years, being male, being born or brought up in a rural area, and having worked before joining medical school (in-service) were significant factors that increased the likelihood of accepting deployment to a rural hospital after graduation.

Our survey showed that male medical students were about three times more likely to accept deployment in a rural area than female. There have been mixed results on gender influence for uptake of rural practice. Thus, for example, while in Ghana it was reported that male gender influenced rural practice choice [25], in Saudi Arabia no difference in preference was observed [26]. In the Tanzanian context, the influence of gender on place of practice choice could much depend on family considerations such as place of employment of the husband (if married) or location of parents whereby rural residence of parents increases the probability of choosing to practice in areas nearer the location of parents [27]. Available literature, however, indicates that the determining factor for women’s job mobility is family considerations, while for men it is economic considerations [7].

Leon and Kolstad [24], in their study in Tanzania among fifth year medical students in three medical schools, found that age above 26 years influenced acceptance of employment in rural settings. In our case, this could be explained by the fact that, most of these students are in-service and therefore have experience with rural settings (most of them being middle cadre clinicians who were deployed in lower level health facilities found in rural areas). This observation was further strengthened after binary logistic regression of rural birth and in-service enrolment on acceptance of a job in a rural hospital, whereby in-service enrolment status remained to be a significant predictor. McDonald et al. [43] found that Australian medical graduates with rural origins were about four times likely to undertake rural practice compared to those with an urban place of birth and/or upbringing. Similar evidence has been documented in several studies [23, 27, 29, 44–46]. Comparable results have been reported in Tanzania whereby it was shown that final year medical students in three medical schools who had not spent their childhood in Dar es Salaam (the biggest city in Tanzania) were more likely to accept a job in a district hospital (mostly found in remote/rural areas), although the study could not establish this difference for students with rural birth perse [21]. The inclination of medical students with rural backgrounds to practice in rural areas after graduation could be explained by their familiarity with rural life and cultural norms [46].

Multivariate logistic regression isolated satisfaction with peripheral hospital rotation and male gender as two independent predictors for willingness to accept a job in rural health facilities. In their study in Kenya, Kibore et al. [37] showed that clinical rotations in peripheral, non-tertiary hospitals motivated medical students to accept employment in those hospitals. The same result was reported in a study in Uganda whereby it was shown that the choice to work in rural and underserved areas was greatly influenced by exposure to community-based training experiences [47]. Somers and Spencer [48] demonstrated that upon graduation, students who had exposure to rural rotations had a significantly higher probability of choosing rural practice compared to those who were not and hence a higher probability of choosing rural practice. Wandiraa and Maniple [9] argued that preference for working in rural areas commences and grows during training, and exposure to rural environments during clinical rotations should cement this preference. However, Leon and Kolstad [24] in their study in Tanzania, found that students who underwent community health rotations during medical training were less likely to accept deployment in a rural hospital after graduation. They attributed this to either general dislike of the rural areas due to poor working environment (poor infrastructure, lack/shortage of medical equipment and supplies etc.) or poor organization and/or content of the community rural rotations. Gum argued that poorly organized rural placement can negatively impact students on their willingness to uptake a job in rural areas [49]. Thus, it is important that good educational environment such as comfortable accommodations [50, 51] adequate allowances and internet access [52] is created to influence students to accept a job in a rural setting.

The higher likelihood of in-service students to accept rural practice could be explained by the fact that most of these were admitted at the medical school after practicing at lower cadre for some time (mostly in rurally-located health facilities), although the difference was only a trend and not significant. Their familiarity with working in rural environments could influence their preferences to continue practice in similar environments. The low number of in-service students in our study could explain the insignificant difference.

### Strengths and limitations of the study

#### Strengths

This study attempted to find out if stand-alone short-term placements during medical training could influence future acceptance of rural practice in a resource-limited setting (RLS). Most of the studies on the role of rural exposure during undergraduate medical training in increasing rural medical practice after graduation have been done in high-income countries and very few, if any, in resource-limited settings. Since health workers' mal-distribution is prevalent in RLS, the results of this study are of great importance.

### Limitations

Our study relied on self-reported data from students with high reliance on preferences rather than actual practice, which could weaken the strength of our conclusions. While the majority of MD3 students completed the survey, not all students participated, and it is possible that the non-participants might have more negative perceptions of peripheral rotations. Also, the lack of a comparison group could make it difficult to understand how the predictors are influencing preferences. Furthermore, the lack of feedback information from the peripheral hospitals to complement students’ self-reported data coupled with other individual and group effects of the surveyed students also limit the strength of our conclusions. A stronger study designs that incorporates a comparison group could lead to better understanding of our results.

## Conclusions

Satisfaction with the rural rotation program was associated with increased likelihood of rural practice after graduation. Opportunities exist in reducing mal-distribution of health care workers through interventions that target health care workers in-training. Institutions of higher learning and other stakeholders involved in human resource for health (HRH) should consider introducing well organized, rural clinical rotations in their medical curricula to increase the likelihood of future rural location placement. Also, recruitment of more in-service students, especially those who have worked in rural areas for at least two years should be considered due to their high propensity to return back to rural areas after graduation.

### Way forward

This was a cross-sectional study with its pertinent shortcomings. Follow-up studies that will utilize national and KCMUCo alumni databases will provide a better analysis of the translation of intent to actual practice and retention in rural and/or underserved areas.
